# Predictors of Quality of Life in Children and Adolescents with Psychiatric Disorders

**DOI:** 10.1007/s10578-019-00914-4

**Published:** 2019-07-23

**Authors:** Dennis Bastiaansen, Robert F. Ferdinand, Hans M. Koot

**Affiliations:** 1 Department of Child and Adolescent Psychiatry/Psychology, Erasmus University Medical Center Rotterdam, Rotterdam, The Netherlands; 2 Yulius Center for Child and Adolescent Psychiatry, Dordrecht, The Netherlands; 3 Department of Child and Adolescent Psychiatry, GGZ Delfland, Delft, The Netherlands; 4 Department of Clinical, Neuro and Developmental Psychology, Vrije Universiteit Amsterdam, Van der Boechorststraat 7, 1081 BT Amsterdam, The Netherlands

**Keywords:** Children, Predictors, Psychiatry, Quality of life

## Abstract

This study aimed to identify factors that predict quality of life (QoL), over and above potential improvements in QoL related to a decrease in psychopathology, in children and adolescents with psychiatric problems. Two hundred and thirty one referred children and adolescents, aged 7–19 years, were followed up across a 1-year period. QoL and psychopathology were assessed, as were a broad range of child, parent, and family/social network factors. Time 1 QoL scores and change in level of psychopathology from Time 1 to Time 2 were important predictors of Time 2 QoL scores. Lower than expected Time 2 QoL was also predicted by the presence at Time 1 of a chronic physical disease, low self-esteem, poor social skills, and stressful life events. Findings can be useful to identify children who are at risk for lower than expected levels of QoL, even after receiving help from mental health agencies.

## Introduction

All clinicians who work with children with psychiatric problems are acquainted with a group of children in which treatment sorts out little improvement in psychopathology. This is in accordance with studies showing that psychopathology in children tends to persist into adolescence and young adulthood [1–3], and that clinical interventions often do not yield complete reduction of psychiatric symptoms [4–6]. Although clinicians often aim to diminish the frequency or severity of psychiatric problems, a major goal of mental health care workers is to improve the quality of life (QoL) of their patients [7]. Since psychopathology tends to persist, it can be hypothesized that QoL does not improve sufficiently even in those who are treated. The need to address QoL in child psychiatric treatment is also shown by studies that indicated that the QoL of children with psychiatric problems is considerably poorer than that of children from the general population, and as poor or even poorer than that of physically ill children [8–10]. If, prior to treatment, it would be known which children have a high probability of less than optimal improvement of their QoL, this might assist clinicians with their treatment considerations.

So far, only two studies addressed predictors of change in QoL over time in a clinical sample of children and adolescents with psychiatric problems. Bastiaansen et al. [11] studied the association between change in psychopathology and QoL across a 1-year period in children and adolescents with high levels of psychopathology at initial assessment. It was found that psychiatric symptom reduction was associated with an improvement of QoL. However, an improvement of QoL was also reported in a number of children with persistently high levels of psychopathology [11], which suggests that QoL may improve, even if reduction of psychiatric symptoms is not achieved. A 3-year follow-up study of adolescents by Jozefiak et al. [12] demonstrated that poorer family functioning at baseline, reported by parents, was significantly associated with worsening QoL during follow-up period. The other factors included, Time 1 adolescent reports of anxious/depressed symptoms and self-esteem, were not associated with change of QoL over the follow-up period. Change in psychopathology was not included as a factor. This raises the question which other factors are associated with significant improvement of QoL in children with psychiatric problems, over and above the potential change in QoL associated with reduction of psychiatric symptomatology. Treatment modules aiming at such factors might help to improve QoL in children with persistently high levels or only limited improvement of psychopathology. If other factors indeed are important for the improvement of QoL this may have implications for the organization and provision of treatment packages that address these factors.

Several personal and environmental factors, that may be grouped according to “proximity” [cf. 13] to the child may predict QoL improvement in children with psychiatric problems. Personal factors include child characteristics, both emotional/behavioral and physical, that are likely to have an immediate effect on the child’s functioning and experience. Examples of these variables are sex and age, or the child’s self-esteem and social skills. The direct environment of the child includes contextual factors directly related to the child’s functioning and development, like parental psychopathology and family functioning. More distal factors exert a potential influence on the child but are not necessarily directly related to or aimed at the child such as social network factors, like social support from friends.

Two cross-sectional studies identified factors that were associated with QoL in children with psychiatric problems [14, 15]. Both studies found a strong association between psychopathology and poor QoL. Besides, QoL was predominantly associated with child factors, such as a chronic physical disease, low self-esteem, poor social skills, temperamental characteristics, and coping style, and also with family/social network factors, such as stressful life events, poor family functioning, and poor social support. These factors may also predict improvement of QoL across time over and above the influence of changes in psychopathology.

In the present study, children and adolescents with psychiatric problems were followed-up across a 1-year period using the same sample as in a previous study [11]. The aim of the study was to investigate which child, parent, and family/social network factors predict QoL at follow-up, over and above potential improvements in QoL related to a decrease in psychopathology. We hypothesized that personal and environmental sources of support, like good social skills and support from family and peers would be associated with a better than expected QoL at follow up, whereas factors indicating heightened levels of stress, like the presence of a chronic physical disease or the occurrence of stressful life events would be associated with lower than expected QoL.

## Methods

### Procedure and Participants

The present sample is a 1-year follow-up study of a child psychiatric outpatient sample. The first assessment (Time 1), addressed a sample of 310 children and adolescents (response rate 73.1%; mean age 11.3 years; range 6–18 years), who had been referred during a 1-year period to a general or a university child psychiatric outpatient department in Rotterdam, The Netherlands. By recruiting patients from these two clinics, children with a broad range of problems, varying from mild to severe, were included [14, 16]. Children and parents filled out questionnaires concerning QoL, psychopathology and a broad range of child, parent, and family/social network factors. The child’s clinician provided information on the DSM-IV diagnosis, family functioning and functional impairment of the child. Teachers filled out questionnaires regarding psychopathology and the child’s social skills. At the second assessment (Time 2), approximately 1 year after Time 1, children and parents were asked for participation again and after they provided written informed consent, questionnaires were sent by mail. Home visits only took place if children or parents were not capable to answer the questionnaires themselves, because of reading difficulties or language problems. In that case, a research assistant helped them to complete the questionnaires at home. Children and parents rated questionnaires concerning QoL and psychopathology. After consent of parents and children, teachers filled out a questionnaire regarding child psychopathology. The study was conducted after approval by the Erasmus MC university hospital medical ethical committee.

At Time 2 (mean follow-up time 389 days; SD = 66 days), 231 children and their parents participated (response rate 74.5%). The mean age of the sample of 134 boys (58.0%) and 97 girls (42.0%) was 12.2 years (SD = 3.2; range 7–19 years). Family socio-economic status (SES) was determined through parental occupational level [17]; 30.7% of the children came from families with low and 69.3% from families with middle to high SES. Based on the main clinical diagnosis, obtained with the DSM-IV Checklist Interview in a standardized way during Time 1 [18], each child was assigned to one of six diagnostic groups: (1) Attention Deficit and Disruptive Behavior Disorders (n = 74, 32.0%), (2) Anxiety Disorders (n = 43, 18.6%), (3) Mood Disorders (n = 25, 10.8%), (4) Pervasive Developmental Disorders (n = 27, 11.7%), (5) Other Disorders (n = 16, 6.9%; including Somatoform Disorder and Enuresis/Encopresis), and (6) No Diagnosis (n = 46, 19.9%). The validity of the Dutch version of the DSM-IV Checklist Interview was supported by Bastiaansen et al. [16].

### Instruments

#### QoL Measures

##### Pediatric Quality of Life Inventory™ Version 4.0 (PedsQL)

The 23-item PedsQL [19] was used to measure the child’s QoL. The PedsQL has a child self-report and a parallel parent proxy-report format. Versions for ages 5–7, 8–12 and 13–18 years were used. The respondent is asked to indicate how much of a problem an item has been for the child during the past month. By formulating the instruction in this way, the informant is not asked to rate the presence of a certain behavior, but if present, its impact on the child’s everyday functioning. A 5-point-Likert scale format is used and scores may range from 0 to 100, from ‘almost always a problem’ to ‘never a problem’; higher scores indicate a better QoL. Four subscales and a Total Score are computed, covering the following dimensions of QoL: (1) physical functioning (8 items; e.g. ‘hard to do sports’ or ‘having hurts’), (2) emotional functioning (5 items; e.g. problems with ‘feeling angry’ or ‘trouble sleeping’), (3) social functioning (5 items; e.g. ‘trouble getting along with peers’ or ‘being teased’), and (4) school functioning (5 items; e.g. ‘trouble keeping up with schoolwork’ or ‘missing school’). In the present study only the Total Score was used, computed as the sum of the 23 items divided by the number of items answered. Good reliability and validity were reported for the American [19] and Dutch version [10] of the PedsQL.

#### Psychopathology Measures

##### Child Behavior Checklist 4–18 (CBCL) and Teacher’s Report Form (TRF)

The CBCL [20] and TRF [21] were used to obtain standardized parent and teacher reports of child psychopathology. The second part of the CBCL and TRF, which were used in the present study, consists of 120 items on behavioral or emotional problems in the past 6 or 2 months for CBCL or TRF, respectively. The child’s behavior is rated on a three point scale (0 = not true, 1 = somewhat true, 2 = very or often true) and summing the scores for each problem item yields the Total Problem Score. Higher scores indicate a higher level of psychopathology. The good reliability and validity of the Dutch CBCL and TRF were supported by Verhulst et al. [22, 23].

#### Child Factors

##### Intelligence

At Time 1 the Wechsler Intelligence Scale for Children—Revised (WISC-R) [24] was used to measure the intelligence of the child. In 12.1% of the children this was not possible, because their IQ was too low to be measured (n = 9) or because their age was above the age range (> 16 years) for which WISC-R norms are available (n = 19). Self-report questionnaires were not obtained from the low IQ children of the first group (n = 9).

##### Chronic Physical Disease

The presence of a chronic physical disease at Time 1 was assessed with the Questionnaire for Identifying Children with Chronic Conditions (QuICCC [25]. This questionnaire consists of 39 items and each item consists of three sequences. The first part of each question sequence asks about one or more specific consequences of having a chronic health condition; the second part asks whether the consequence is the result of a medical, behavioral, or other health condition; and the final part assesses the duration of the condition, which has to be at least 1 year. To meet the definition of a chronic disease, a child must qualify in each component of at least one question sequence. Good reliability and validity of the QuICCC have been demonstrated [25].

##### Self-Esteem

To measure self-esteem of the child at Time 1, the Global Self-Worth Scale of either the Self-Perception Profile for Children (SPPC; ages 8–12) [26] or the Self-Perception Profile for Adolescents (SPPA) [27], was used, consisting of 6 or 5 four-point items, respectively. High scores indicate high self-esteem. Good reliability and validity of the Global Self-Worth Scale of the SPPC and SPPA have been reported [26–28].

##### Social Skills

At Time 1 children’s social skills were rated by parents and teachers on separate informant and age versions (6–12 or 13–18 years) of the Social Skills Rating System (SSRS) [29]. Parent forms contain 38 and 40 items for ages 6–12 and 13–18 years, respectively; teacher forms contain 30 items for both age groups. In the present study the Total Score was used, calculated by summing the scores of each individual item; higher scores represent better social skills. Good reliability and validity of the SSRS were reported [29].

#### Parent Factors

##### Psychopathology in Mothers

The Young Adult Self-Report (YASR) [30] was filled out by mothers to assess psychopathology of the mother at Time 1. The YASR has the same format as the CBCL and concerns the past 6 months. Only the 29 problem items that best discriminated between referred and non-referred subjects were used [31]. A Total Problem Score was computed by summing the scores on the 29 items. Good reliability and validity of the Dutch YASR have been demonstrated [32].

##### Psychiatric Treatment of the Mother

At Time 1, current and past inpatient and outpatient mental health care use by the mother was assessed with a questionnaire on mental health use.

##### Parenting Stress of the Mother

The Nijmegen Parenting Stress Index (NPSI [33] was completed by mothers at Time 1, which is a modified Dutch version of Abidin’s Parenting Stress Index [34]. This questionnaire measures the level of perceived parenting stress of the mother originating from several child and parent characteristics within the caregiving context. A short form of 25 items, derived from scales measuring the perceived child and parent characteristics was used [35]. A Total Problem Score was computed by summing the scores on the 25 items.

#### Family/Social Network Factors

##### Family SES

At Time 1 was assessed through parental occupational level [17]. Based on the highest occupational level in the family (father or mother), the family was assigned to one of two categories: low SES or middle-high SES.

##### Family Functioning

The two caregiver resources scales of the Child and Adolescent Functional Assessment Scale (CAFAS) [36], indicating whether the caregiver meets the child’s material needs and social support, were rated by the child’s clinician at Time 1. These scales have been designed to assess functional impairment in the family. A Total Score is computed as the sum of the scores on the two scales. Scores were recoded (ranges 0–60) so that higher scores indicate better caregiver functioning. The CAFAS was found to be a reliable and valid instrument [37].

Parents filled out the General Functioning Subscale of the McMaster Family Assessment Device (FAD [38]. This scale measures the overall health/pathology of the family. Items were scored in such a way that higher scores indicate better family functioning. Good reliability and validity of the General Functioning Subscale have been reported [38].

##### Social Contacts of the Family

To measure the social contacts of the family at Time 1, parents filled out the Health Insurance Experiment Social Support Questionnaire [39], concerning social contacts and social resources of the family. It contains nine items and summing these items yields an Overall Social Contacts Index. Higher scores indicate more social contacts.

##### Social Support

To assess child-perceived support from significant others at Time 1, children completed the Social Support Scale for Children (SSSC; ages 8–12) [40] or the Social Support Scale for Adolescents (SSSA; ages 13–18) [41]. These questionnaires originally consist of four scales and in this study three scales were used, measuring social support from family members, friends, and classmates. Each subscale consists of six items and Harter’s four-point item format was used for the classmates’ scale; the family members and friends scales were slightly changed into a two-point format. Higher scores indicate greater perceived support. Harter reported good reliability and validity for the SSSC and SSSA [40, 41].

##### Life Events

At Time 1 parents completed a 12-item short form of the Life Events Questionnaire [42], which has a ‘yes’ or ‘no’ format and assesses potentially stressful life events such as parental divorce, death of a family member, or long-term hospitalization in the past 2 years. Item scores are summed into a Total Life Events score; higher scores indicate more life events. A satisfactory reliability of the LEQ has been reported [42].

### Statistical Analyses

To estimate possible predictors of Time 2 child and parent reported QoL scores, multiple linear regression analyses were conducted. To identify predictors of child reported QoL, Time 2 PedsQL child report Total score was entered in a regression analysis as the dependent variable, and candidate predictors were entered as independent variables in subsequent blocks (method Enter). Before each next block was added into the analysis, non significant predictors in the previous block were removed from the model. In the first block, the Time 1 PedsQL child report Total score, standardized residual scores of CBCL and TRF, together with sex and age and interaction terms of PedsQL, CBCL, and TRF with sex and age, were entered as independent variables. The standardized residual scores of CBCL and TRF were computed by regressing Time 2 CBCL and TRF scores on Time 1 CBCL and TRF scores, respectively, in order to assess effects of change in level of psychopathology from Time 1 to Time 2. In the second block of the analysis, the Time 1 child factors (intelligence, presence of psychiatric diagnosis, presence of a chronic physical disease, self-esteem, and social skills) were added, together with interaction terms between these factors and sex and age, to test if these factors improved the prediction of QoL. Parent factors were entered in the third block (psychopathology mother, maternal mental health use, and parenting stress), also together with interaction terms between these factors and sex and age. Finally, family/social network factors were entered in the fourth block (family composition, SES, family functioning, social contacts of the family, perceived social support, and stressful life events), simultaneously with interaction terms between these factors and sex and age.

To identify predictors of parent reported QoL, a similar analysis was performed, in which Time 2 PedsQL *parent* report Total score was entered as the dependent variable and Time 1 PedsQL *parent* report Total score as independent variable.

## Results

### Descriptive Analyses

Table 1 shows the means, standard deviations, and ranges of QoL and child psychopathology measures at Time 1 and Time 2 and of predictor variables at Time 1. Total scores on child and parent versions of the PedsQL were higher at Time 2 than at Time 1 (*p *< 0.001 for both PedsQL child and parent report; paired-sample *t* tests), which indicated improvement in QoL across time. Scores on the CBCL and TRF decreased across time (*p *< 0.001 for CBCL and *p *< 0.005 for TRF, respectively; paired-sample *t* tests), indicating improvement in psychopathology across the 1-year period.

**Table 1:** Means/percentages (SD), and ranges of QoL and child psychopathology measures at Time 1 and Time 2 and of predictors at Time 1 (N = 231)

Measures	Instrument	Mean/percentage	Range	Alpha
QoL				
Time 1				
QoL total score—child report	PedsQL	71.7 (12.9)	35.9–98.9	0.84
QoL total score—parent report	PedsQL	66.1 (14.2)	27.2–97.8	0.87
Time 2				
QoL total score—child report	PedsQL	74.8 (14.2)	25.0–1.00	0.87
QoL total score—parent report	PedsQL	72.9 (15.4)	14.1–1.00	0.87
Child/adolescent psychopathology				
Time 1				
Psychopathology—parent report	CBCL	64.7 (28.1)	6–137	0.90
Psychopathology—teacher report	TRF	50.8 (31.9)	3–147	0.95
Time 2				
Psychopathology—parent report	CBCL	50.1 (28.1)	2–142	0.95
Psychopathology—teacher report	TRF	44.6 (29.3)	4–144	0.97
Child factors				
Intelligence	WISC-R	96.4 (15.7)	48–141	
Chronic physical disease (yes)	QuICCC	9.2%		
Psychiatric diagnosis (yes)	DSM-IV CI	80.1%		
Self-esteem	SPPC/SPPA	3.1 (0.7)	1.3–4.0	0.74/0.82
Social skills—parent report	SSRS	41.7 (11.9)	1–73	0.90
Social skills—teacher report	SSRS	31.1 (11.1)	5–57	0.91
Parent factors				
Psychopathology mother	YASR	9.9 (8.6)	0–48	0.92
Psychiatric treatment mother (yes)		35.0%		
Parenting stress mother	NPSI	83.1 (26.7)	26–137	0.95
Family/social network factors				
Single parent family (yes)		25.5%		
SES^a^		69.3%		
Family functioning—clinical report	CAFAS	53.8 (8.2)	20–60	0.67
Family functioning—parent report	FAD	3.1 (0.5)	1.5–4	0.87
Social contacts family	HIESSQ	3.0 (0.8)	0.7–5	0.73
Social support—family	SSSC/SSSA	0.7 (0.2)	0–1	0.80/0.79
Social support—friends	SSSC/SSSA	0.8 (0.2)	0–1	0.76/0.82
Social support—classmates	SSSC/SSSA	3.2 (0.7)	1–4	0.71/0.68
Stressful life events	LEQ	1.5 (1.3)	0–6	

^a^Dichotomized variable: SES (low = 0 versus middle-to-high = 1). Alpha = Cronbach’s alpha

### Multiple Regression Analyses

#### PedsQL Child Report

The final model of PedsQL child report was significant at *p *< 0.001 (F(7, 144) = 19.4). The R^2^ of this model was 0.49, which means that 49% of the variance in Time 2 PedsQL child report Total score was explained by the predictors included in the model (large effect size according to Cohen [43]).

The Time 2 PedsQL child report Total score was predicted by the Time 1 PedsQL child report Total score and change in level of child psychopathology (CBCL). Over and above the improvement in QoL related to improvement in psychopathology, other factors also contributed independently to the variance in the regression model.

Chronic physical disease at Time 1 was an important predictor of the Time 2 PedsQL child report score; if a physical disease was present at Time 1, the PedsQL child report score at Time 2 was significantly lower. The analysis also learned that the impact of a physical disease on Time 2 QoL was larger for younger children than for older children, because younger children with a physical disease at Time 1 had a lower QoL score at Time 2. Self-esteem and social skills (parent report) also predicted PedsQL child report scores at Time 2. The significant effect of the interaction between sex and self-esteem on Time 2 PedsQL child report, indicated that an increase in QoL associated with higher levels of self-esteem was larger for boys than for girls. Higher levels of social skills at Time 1 also predicted a higher QoL score at Time 2. The child factors chronic physical disease, self-esteem and social skills together added 8% variance to the model. Remarkably, no parent or family/social network factors predicted child reported QoL scores at Time 2.

Table 2 displays standardized beta coefficients of the predictors. Since these are standardized (z-score) values, the importance of the different predictors can be compared, because they are all in the same unit of measurement. The standardized beta coefficient of Time 1 QoL Total score was 0.41 and it seems to be the most important predictor of Time 2 PedsQL child report Total score. The range of the standardized beta coefficients of the other predictors was between 0.16 and 0.29 and it may be concluded that the contribution of each of these factors to the variance of the regression model is approximately equal.

**Table 2:** Multiple linear regression of Time 2 QoL on change of psychopathology and Time 1 predictors

Predictor variables	Instrument	Time 2 outcomes	
		PedsQL total child report	PedsQL total parent report
Block 1 (Time 1 QoL score and psychopathology change)			
*R*^2^		0.40	0.58
Time 1 QoL Total score	PedsQL	0.41	0.50
Change in child psychopathology (parent report)	CBCL	− 0.20	− 0.52
Block 2 (child factors)			
*R*^2^ change		0.09	–
Chronic physical disease	QuICCC	− 0.29	
Self-esteem	SPPC/A	0.19	–
Social skills (parent report)	SSRS	0.16	–
Sex^#^ × self-esteem^a^	Sex × SPPC/A	− 0.26	–
Age^#^ × chronic physical disease^b^	Age × QuICCC	0.21	–
Block 3 (parent factors)		–	–
Block 4 (family/social network factors)			
*R*^2^ change		–	0.05
Sex^#^ × stressful life events^c^	Sex × LEQ	–	0.24
*R*^2^ for the full model		0.49*	0.63**

Entries are standardized betas for the full model; only significant betas *(p *< 0.05) are shown

*F(7, 144) = 19.4, *p *< 0.001

**F(5, 165) = 55.6, *p *< 0.001

^#^Dichotomized variables: sex [boy (0) vs. girl (1)] and age [8–12 (0) vs. 13–18 (1) years]

^a^The increase in QoL with an increase of self-esteem was larger for boys than for girls

^b^Younger children with a physical disease had a lower QoL than older children

^c^The decrease in QoL with an increase of stressful life events was larger for boys than for girls

#### PedsQL Parent Report

The final model for the PedsQL parent report was significant at *p *< 0.001 (F(5, 165) = 55.6) and the R^2^ of this model was 0.63, which means that 63% of the variance in Time 2 PedsQL parent report Total score was explained by the predictors included in the model (large effect size according to Cohen [43]).

The Time 2 PedsQL parent report Total score was predicted by the Time 1 PedsQL parent report Total score and change in level of child psychopathology (CBCL). In contrast with the regression model for the PedsQL child report, no child or parent factors predicted parent reported QoL at Time 2. However, of the family/social network factors, the interaction term of stressful life events and sex predicted Time 2 PedsQL parent report. Stressful life events at Time 1 predicted a lower Time 2 PedsQL parent report and the impact of stressful life events on QoL appeared to be larger for boys than for girls, because boys who experienced stressful life events before Time 1 had lower Time 2 PedsQL parent report scores than girls who experienced stressful life events. This predictor added 4% variance to the model, over and above the variance of Time 1 QoL score and change in level of psychopathology.

Comparison of the beta coefficients for the predictive relation between Time 2 parent reported QoL and Time 1 parent reported QoL and parent reported Time 1 – Time 2 change in psychopathology showed that both factors had a similar weight in the regression equation. This indicates that QoL at intake and change of psychopathology are equally influential in predicting QoL 1 year later.

## Discussion

The present 1-year follow-up of children and adolescents with psychiatric problems investigated which child, parent, and family/social network factors predict QoL at follow-up, over and above potential improvements in QoL related to a decrease in psychopathology. In addition to the Time 1 QoL score, change in level of psychopathology from Time 1 to Time 2 appeared to be an important predictor of Time 2 QoL score in both child and parent report on QoL. Besides, other factors including Time 1 reported chronic physical disease, level of self-esteem, level of social skills and presence of stressful life events also contributed independently to the variance in the regression models based on child and parent report on QoL.

Presence of a physical disease has previously been reported to be associated with QoL in a study with a cross-sectional design [14], but not yet as a predictor of QoL over time in children with psychiatric disorders. Although several studies found that a physical disease decreases QoL of children at large [44–46], this study showed that the presence of a physical disease has an additional diminishing effect on the QoL of children with psychiatric problems. For clinical practice this implicates that extra attention should be paid to an adequate treatment of a co-current physical disease in children with psychiatric problems and that attention should be paid to the functional impairments due to the physical disease. This is especially important in younger children since our results showed that the cross-time influence of physical disease on QoL was stronger for younger than for older children.

Various reviews support the view that comorbidity does not simply mean the addition of two diseases that independently follow their usual trajectories. Instead, the simultaneous presence of two diseases may lead to an increasing number of complications, and make the treatment of both diseases more difficult and, possibly, less efficacious [47]. Unfortunately, despite considerable comorbidity rates between physical and mental disorders Merikangas et al. [48], treatment of psychiatric disorders often is organized separately from interventions for physical conditions, and vice versa. The present study underscores the urge for integrated interventions, targeting both physical and mental diseases, and the need to lower walls between psychiatry and other medical disciplines, to improve treatment outcome, and to ameliorate QoL.

Self-esteem was another predictor of Time 2 QoL score. It appeared that the positive effect of self-esteem on level of QoL was larger for boys than for girls. In the study of Bastiaansen et al. [14] self-esteem has already been reported to be associated with QoL, but the present study showed that self-esteem might also be a predictor of QoL, although this findings was not confirmed by the Jozefiak et al. study [12]. The findings suggest that strengthening the self-esteem of children with psychiatric problems may help to enhance their QoL over time. Some treatment programs already aim at enhancing self-esteem (e.g. [49]), but the effect on QoL of such interventions has not been studied yet.

Better social skills at Time 1 predicted a higher QoL 1 year later. No sex or age differences were found. An association between poor social skills and poor QoL has been reported previously [14]. The present study underscores the possible importance of social skills training, that is already implemented in some treatment programs [50, 51]. Self-esteem and better social skills predicted higher QoL at Time 2, independent of the course of psychopathology. This may indicate that self-esteem and social skills should be considered as generic risk factors for poor QoL, that should be addressed separately from psychopathology. Screening of, and if necessary, treatment of self-esteem and social skills may be important in all children who are referred for mental health services, besides diagnostic evaluation for psychiatric diagnoses.

Apparently, child characteristics reflective of competence, including self-esteem and social skills, may influence the child’s well-being despite the presence of psychiatric problems and independent of the degree of improvement of these problems. This influence may work along at least two ways. First, the effect of psychopathological problems on problems experienced in major areas of functioning may be buffered by feelings of high competence as well as ascertained skills in social interactions with peers and adults. Based on these competencies these children may be more active and effective than other children with similar levels of psychopathology. Alternatively, these children may be positively biased in reporting on their QoL. However, this seems less likely given that the parents reported on social skills.

Remarkably, no parent or family/social network factors predicted the Time 2 PedsQL child report Total score. Apparently, QoL as reported by the child him/herself, is best predicted by factors proximal to the child. This hypothesis is supported by the fact that not all child factors were reported by the child him/herself, e.g. social skills were reported on by the parent, but still was significant in the final model.

Time 2 PedsQL parent report Total score was also predicted by the presence of stressful life events before Time 1. Apparently boys are more sensitive to this influence, since the decrease in QoL with an increase of stressful life events was larger for boys than for girls. Presence of stressful life events has previously been reported as a factor associated with QoL in studies with a cross-sectional design [14] and as a predictor of change in psychopathology [52]. The current results show that stressful life-events not only influence a child’s QoL through their association with child psychopathology but also predict QoL more directly.

The current study adds to earlier findings that children with psychiatric problems are at risk for reduced QoL at least as much as children with chronic health conditions [9, 10] by demonstrating that other child and environment related factors may contribute to its improvement. In future studies, it may be important to address the differential impact of psychiatric disorders, although like with physical conditions it may be best to use a non-categorical approach, as the commonalities across disease groups far outweigh differences (cf. [53]). Further, it should be noted that children with chronic disease are at increased risk for psychopathology [54] and that some of the impact of psychiatric problems on QoL may be mediated through the impact of functional limitations due to associated physical conditions.

### Limitations and Implications

This study is not without limitations. A first possible limitation might be the duration of the follow-up period. Children were followed-up across a 1-year period. It might be possible that this period is too short to detect clinically significant change in level of QoL in some children and also to identify predictors of QoL across a follow-up period. Longer follow-up periods may be needed to evaluate the potential effect of psychiatric treatment on the course of QoL for those who have been treated as well as the influence of other factors that only becomes visible after some time.

Secondly, since the nature of the present study was explorative, and treatment was unstandardized, our study gives no insight in the effects of treatment and the generalizability of the current findings across other treatment settings is hampered. Future studies might address the influence of treatment as well as of its interaction with child, family, and environmental on change in child QoL.

Thirdly, this study used both child and parent reports of psychopathology, QoL, and predictor constructs. Although QoL is an intrinsically subjective construct [55] and informant agreement is generally only moderate at best, both for QoL and psychopathology reports (e.g., [56, 57]) we chose both informants as multiple informants may add different but reliable and valid information, especially when children under 11 are involved.

Fourth, although the sample was drawn from children and adolescents referred for specialist psychiatric services a sizeable proportion did not receive a psychiatric diagnosis despite high levels of psychopathology. Although no diagnostic category was assigned these children suffered from sufficient symptoms and functional limitations to justify psychiatric treatment as established during intake. Importantly, presence of diagnosis did not have a significant contribution in the analyses.

This study’s findings have several implications for assessment, interventions, service costs, treatment reviews, and concurrent or additional involvement of other agencies. They call for broad assessment of several different functional domains next to psychiatric symptomatology and QoL indicators as these may all be important for treatment as well as treatment evaluation. The study also taps into a change in which psychiatric services have become more demand oriented than supply oriented. While earlier all sorts of standard diagnosis-treatment modules were offered emphasis is now more on the initial help question from which clear treatment goals are formulated for a set period of time. Moreover, more evaluation moments are now included in order to reduce highly specialized services and scale services down to more basic levels. Also, more often other services (e.g. social work, school psychological services) are invoked to offer guidance at home or school. These services are much less symptom oriented and focus more on several different functional domains. The findings of this study suggest domains to be addressed in these services, and that these services are worth their cost as they add to the children’s QoL. They also suggest the importance of liaisons between psychiatric and pediatric services as indicated above. Finally, they suggest that treatment reviews need to address QoL and other functional domains next to reduction of symptoms of the diagnosed disorder.

## Conclusion

The present study assessed predictors of QoL across time in children with psychiatric problems, using information about different domains of functioning and from multiple informants. Change in psychopathology was clearly related to improvement in both child- and parent-rated QoL. Therefore, reduction of psychopathology seems to remain crucial to improve children’s well-being. It was shown that, besides psychopathology, other factors have their own specific contribution to future QoL. Early identification of these factors may assist in the development of early intervention of poor QoL in clinical practice. Mental health professionals must be able to intervene and follow up with children who are at high risk of poor QoL in order to provide them with adequate services and prevent the development of additional problems.

## Summary

A major goal of mental health care workers is to improve the QoL of their child and adolescent patients. However, since psychopathology tends to persist, QoL may not improve sufficiently even in those who are treated. If, prior to treatment, it would be known which children have a high probability of less than optimal improvement of their QoL, this might assist clinicians with their treatment considerations. This study aimed to identify factors that predict QoL across a 1-year period, over and above potential improvements in QoL related to a decrease in psychopathology, in children and adolescents with psychiatric problems. Two hundred and thirty-one referred children and adolescents, aged 7–19 years, were followed up across a 1-year period. QoL and psychopathology were assessed, as were a broad range of child, parent, and family/social network factors. Information was obtained from children, parents, and teachers. Time 1 QoL scores and change in level of psychopathology from Time 1 to Time 2 were important predictors of Time 2 QoL scores. However, lower than expected Time 2 QoL was also predicted by the presence at Time 1 of a chronic physical disease, low self-esteem, poor social skills, and stressful life events. Findings can be useful to identify children who are at risk for lower than expected levels of QoL, even after receiving help from mental health agencies. This may help clinicians to identify children and families who may need additional help.
